# Advances in β-Galactosidase Research: A Systematic Review from Molecular Mechanisms to Enzyme Delivery Systems

**DOI:** 10.3390/pharmaceutics17121538

**Published:** 2025-11-29

**Authors:** Márton Király, Ádám Tibor Barna, Nikolett Kállai-Szabó, Borbála Dalmadiné Kiss, István Antal, Krisztina Ludányi

**Affiliations:** 1 Department of Pharmaceutics, Semmelweis University, Hőgyes E. Street 7-9, 1092 Budapest, Hungary; kiraly.marton@semmelweis.hu (M.K.); barna.adam@semmelweis.hu (Á.T.B.); kallai.nikolett@semmelweis.hu (N.K.-S.); kiss.borbala@semmelweis.hu (B.D.K.); ludanyi.krisztina@semmelweis.hu (K.L.); 2 Center for Pharmacology and Drug Research & Development, Semmelweis University, 1085 Budapest, Hungary

**Keywords:** β-galactosidase, lactase, enzyme formulations, enzyme replacement therapy, lactose intolerance, lactose

## Abstract

**Background/Objectives:** β-galactosidase (lactase) is a transformative enzyme used in many different fields. Its significance spans from biotechnology to food and pharmaceutical industries. β-galactosidase catalyzes the hydrolysis of lactose into glucose and galactose. In medicine, β-galactosidase has gained attention and has many applications, mainly in enzyme replacement therapy. β-galactosidase is the main active ingredient of medications for lactose intolerance. Industrially β-galactosidase is typically produced by the *Aspergillus oryzae* filamentous fungus. Therapeutic interventions involving β-galactosidase aim to mitigate symptoms and improve the patients’ quality of life. In the food industry, it plays a crucial role in the production of lactose-free products, improving accessibility to dairy products. However, despite its versatility and wide use, challenges connected to β-galactosidase still exist, such as the need for cost-effective and more efficient methods for administering the enzyme. Additionally, there are several ongoing studies that seek to enhance stability and optimize the performance of β-galactosidase in various applications. The aim of this manuscript is to summarize current knowledge about β-galactosidase as an active ingredient and to present some preparations that are commercially available or mentioned in the literature. **Methods:** A systematic search was conducted in PubMed, Scopus, Embase and Web of Science to identify relevant articles on formulations related to β-galactosidase, focusing on original research articles published between 1895 and 2025 that exclusively examine the use of oral drug delivery. **Results:** After a rigorous search across multiple databases, 45 relevant studies out of 1633 initial results were selected for analysis. **Conclusions:** β-galactosidase remains a highly versatile enzyme with broad industrial and medical relevance. While current formulations offer significant benefits, further innovation is needed to improve delivery efficiency, stability, and cost-effectiveness. The findings of this review contribute to a deeper understanding of β-galactosidase as an active ingredient and outline opportunities for advancing its application in oral drug delivery systems.

## 1. Introduction

Biological drugs produced by biotechnology are being used with growing frequency. Enzymes are biological catalysts that drive life-sustaining biochemical reactions and have long been acknowledged as critical for maintaining homeostasis and orchestrating complex processes at the cellular level [1]. The intrinsic specificity, catalytic efficiency, and functional versatility of enzymes have led to growing interest in their potential applications in therapeutic interventions. Enzyme-based therapies are rapidly advancing the boundaries in medical science, offering promising avenues for developing targeted treatments with improved efficacy and fewer side effects [2]. This scientific review elucidates the fundamental principles and emerging applications of β-galactosidase-based therapies, highlighting its impact on modern medicine.

β-Galactosidase, also known as lactase, is a globally important enzyme with applications spanning the food, pharmaceutical, and biotechnology sectors. It plays a central role in hydrolyzing lactose, addressing lactose intolerance, a condition affecting over 70% of adults worldwide, particularly in Asia, Africa, and Latin America [3,4]. Beyond its clinical significance, the enzyme is essential in the production of lactose-free dairy products, reducing industrial waste and supporting biofuel generation from whey. While traditionally valued for food and medical uses, emerging research highlights its potential in enzyme engineering, sustainable production, and innovative therapeutic strategies such as prodrug activation. The growing demand for improved formulations and multifunctional applications underscores the need for an integrated understanding of β-galactosidase at molecular, industrial, and clinical levels. Although β-galactosidase is extensively utilized, existing reviews often focus on specific aspects in isolation, providing limited comparative analysis of enzyme sources, formulation strategies, and clinical applications. Additionally, recent advances, like improved stabilization methods and their potential in prodrug activation, are frequently underrepresented. The development and production of biological medical products, or biologics, is becoming increasingly important in the pharmaceutical industry. In 2024, eight of the top ten highest-grossing drugs were protein or peptide types: Keytruda (pembrolizumab), Eliquis (apixaban), Ozempic (semaglutide), Dupixent (dupilumab), Skyrizi (Risankizumab), Darzalex (daratumumab), Mounjaro (tirzepatide), and Stelara (ustekinumab) [5,6]. Biologics can be divided into three main categories: monoclonal antibodies, receptor modifiers and enzymes. Research into all categories is progressing rapidly [7]. Most of the attention is on research into new more usable enzymes, but the development of new drug delivery systems is also important. The primary difficulties include the delivery of enzymes to specific target sites in the body, enzyme denaturation (e.g., from heat/pH extremes, reducing activity by 50% above 50 °C in non-fungal sources), immobilization efficiency (e.g., only 60–80% retention in nanoparticle carriers due to leaching), batch-to-batch variability (arising from fermentation inconsistencies, leading to 10–20% activity fluctuations), their risk for immunogenicity, their instability, the need for frequent dosing, and the expensive production costs limiting their accessibility for patients [8]. To address these challenges, researchers are exploring various potential solutions. With the help of ongoing studies and innovations, these solutions may lead the way for more effective and accessible enzyme therapies in the future [9,10,11]. The objectives of this study are to provide a systematic review of β-galactosidase’s molecular mechanisms and pharmaceutical interventions; evaluate current formulations, including commercial products and innovative delivery systems; discuss challenges, therapeutic potentials, and sustainability aspects; and propose forward-looking strategies for optimization via enzyme engineering and synthetic biology.

### 1.1. Applications for Enzyme Therapies

**Figure 1:**
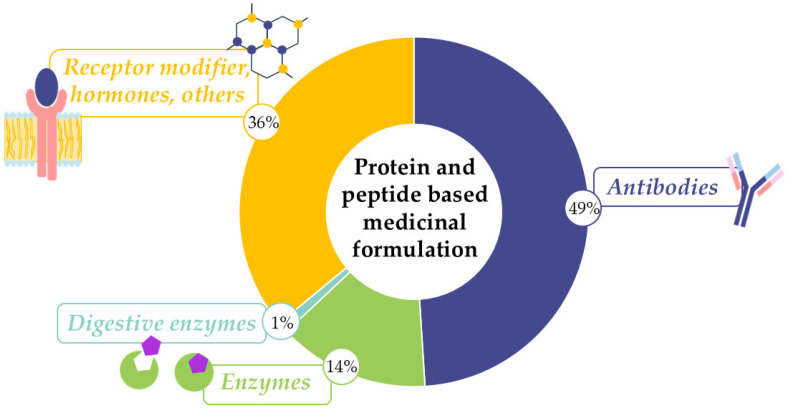
The share of different protein- and peptide-based medicinal formulations, according to Drugbank’s online database: [13].

Enzyme therapies, a large category of protein-based treatments, are applicable to a wide range of diseases, including genetic disorders, cancer, metabolic diseases, and immune-mediated conditions, showcasing remarkable potential (Figure 1) [12].

**Figure 2:**
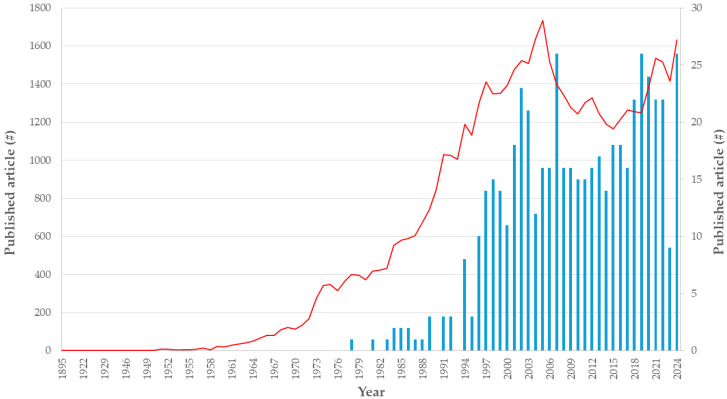
The yearly relevance (number #) of the articles about the β-galactosidase enzyme, using the keywords “(((“beta-galactosidase”) OR (lactase)) OR (“β-galactosidase”))” in blue and “(((“beta-galactosidase”) OR (lactase)) OR (“β-galactosidase”)) AND (formulation)” in red with the Scopus search engine.

Antibody-containing products are the most prevalent, followed closely by different hormones. From all enzyme-type drugs used, the primary digestive enzymes, amylase, lipase, protease and lactase, make up a relatively minor proportion of the total, indicating a vast array of enzymes used in medicine. The relevance of enzyme-containing formulations is expected to increase significantly in the coming years (Figure 2) [14].

Enzyme-based therapies also can be categorized by the therapeutic areas in which they are applied. The most prominent fields include cancer treatment, fibrinolysis, local treatments, and enzyme replacement therapies. In cancer treatment, proteolytic enzymes, such as proteases, are used to selectively degrade proteins, thus hindering tumor growth and metastasis [11,15,16].

Replacement therapies can be categorized into two main types: Systemic and non-systemic. Systemic therapies generally involve administering the enzyme via various injections, enabling it to reach all affected organs and tissues [17]. On the other hand, non-systemic medicines target specific organs or tissues directly, such as administering the enzyme to joints for arthritis treatment or using oral formulations for conditions like lactose intolerance [18]. The development of protein-based formulas is a complex biotechnological process that includes the production, purification, and formulation of the enzyme into a stable and biological drug. Recombinant DNA technology is often employed in manufacturing. After production, the enzyme undergoes purification to remove impurities and is then formulated into a drug product, which can often be administered orally. These pharmaceutical products are available in many forms, including liquid formulations (such as drops or solutions), lyophilized powders, and solid oral forms like capsules, tablets, or chewable tablets [19]. The enzyme can be immobilized on a carrier to improve stability and ensure effective delivery to the target tissue. Various carriers, including hydrogels, liposomes, and nanoparticles, are used for this purpose. Both inorganic (such as silica, iron, and titanium oxide) and organic nanoparticles (such as natural and synthetic polymers like polyvinyl alcohol (PVA), polylactic acid (PLA), chitosan, and polyaspartamides) can be employed in enzyme therapy formulations. Additionally, carbon-based formulations, such as graphene and carbon nanotubes, are also utilized [11,20].

To treat various enzyme deficiency disorders, enzyme-containing pharmaceuticals are used. Pancreatic dysfunctions are addressed with lipase, amylase, and protease enzymes, while β-galactosidase preparations are used to treat lactose intolerance. Galactosidase enzymes, such as lactases that catalyze the breakdown of galactosyl bonds [21], are widely utilized both in the food and pharmaceutical industries due to the increasing prevalence of intolerance to dairy products, which is caused by lactase enzyme deficiency. This metabolic disorder can lead to a severe decline in quality of life [22]. The treatment of this condition is possible with various lactase-containing medications, which are presented separately in a later chapter of this manuscript. Furthermore, since all age groups are affected and the incidence of the condition may exceed 70% of the global population [4,23], it is crucial to find a solution for the proper admission of these types of products that is suitable and easily accessible for everyone. When formulating a pharmaceutical product, developers today must consider factors beyond just the physical–chemical properties of the active and excipient substances. One of the main considerations nowadays is enhancing patient compliance and drug and dosage form stability.

### 1.2. Lactase-Based Therapies and Significance of Lactose Intolerance

Lactase plays a vital role in digestion, producing glucose and galactose as end products of the reaction it catalyzes. These monosaccharides provide energy for the body, which is especially significant during infancy when breast milk is the primary source of nutrition. Therefore, the enzyme is produced in large quantities in newborns and children. Congenital lactase deficiency is an autosomal recessive disorder caused by a mutation in the gene coding for lactase, making it impossible to feed these infants breast milk or any lactose-containing milk or formula [24].

Lactase production decreases after childhood [4]. The ability to properly digest lactose varies geographically, generally decreasing from north to south across the globe, with significant differences observed even among populations of different countries. Consequently, a substantial portion of the adult population lacks sufficient lactase activity, leading to unpleasant gastrointestinal side effects from milk consumption. Most often, β-galactosidase deficiency, also known as lactase deficiency, is the underlying cause of lactose intolerance [25].

Lactose intolerance arises because lactose, unlike monosaccharides (i.e., glucose and galactose), is poorly absorbed in the small intestine. Individuals who are lactase-nonpersistent (do not produce the enzyme at all) cannot digest lactose. Poorly absorbed lactose osmotically attracts fluid, retaining water in the digestive system, which leads to increased intestinal content and diarrhea. Additionally, lametabolizes as a substrate for colonic bacteria, which metabolizes to produce volatile fatty acids and gases such as carbon dioxide, hydrogen, and methane. These gases cause bloating, abdominal pain, nausea, and cramps. Symptoms may not be limited to the gastrointestinal tract; neurological symptoms like headaches and dizziness can also occur [4]. Some cases of infantile colic are also lactose-induced conditions [26].

There are two main types of lactase deficiency: primary (congenital) and secondary (acquired). Primary lactase deficiency is caused by polymorphisms in the transcriptional promoter region of the (lactase coding) *LCT* gene. This congenital enzyme defect is the most common cause of lactase deficiency, affecting one-third of the world’s population. Secondary lactase deficiency can develop as a result of diseases affecting the small intestine [27]. Causes include viral diseases like rotavirus or the loss of the intestinal mucosa after gastrointestinal surgery or celiac disease. If the mucosal surface regenerates and there is no genetic predisposition, lactose digestion may partially or completely recover after healing [28].

The term “classic lactose intolerance” was traditionally used for symptoms that appear after consuming a large amount of lactose but should not be confused with lactase digestion disorder, as intolerance does not always lead to a digestion disorder. Symptom onset depends on the amount of lactose consumed and the transit time of undigested disaccharides reaching the intestine [29].

Symptoms can be alleviated by reducing the amount of lactose intake, such as avoiding certain dairy products. Generally, the amount of lactose in one cup of milk (prox. 12.5 g/250 mL) does not cause symptoms for most people with lactase deficiency. Symptoms become more pronounced when the intake exceeds 18 g and consuming more than 24 g usually results in significant symptoms. The maximum tolerable amount can vary if lactose is consumed with other nutrients. Consuming lactose with meals, especially with fat, slows gastric emptying, reducing the amount of lactose reaching the small intestine per unit time. Certain foods, such as coffee or spicy foods, accelerate lactose transit to the small intestine, thereby increasing symptoms [30]. Fermented dairy products contain less lactose per volume, as lactic acid bacteria in yogurt reduce lactose content through their metabolism [31]. Other factors can also alter gastric emptying or transit time, such as certain medications, diseases, or pregnancy. Lactose-free dairy products are typically sweeter than regular products because glucose and galactose together are sweeter than lactose. They are produced by adding β-galactosidase during the manufacturing process, often making these products more expensive [32].

Beyond lactose intolerance, β-galactosidase shows promise in cancer therapy via prodrug activation systems like Antibody-Directed Enzyme Prodrug Therapy (ADEPT). In ADEPT, β-galactosidase is conjugated to tumor-targeting antibodies, localizing at cancer sites to cleave non-toxic prodrugs (e.g., galactosyl-doxorubicin) into active cytotoxins, minimizing systemic side effects. Studies demonstrate enhanced tumor selectivity and efficacy in models of colorectal and breast cancers, with ongoing research addressing enzyme stability and immunogenicity. This expands its role from digestive aid to targeted oncology [33].

### 1.3. Lactose as Pharmaceutical Excipient

**Figure 3:**
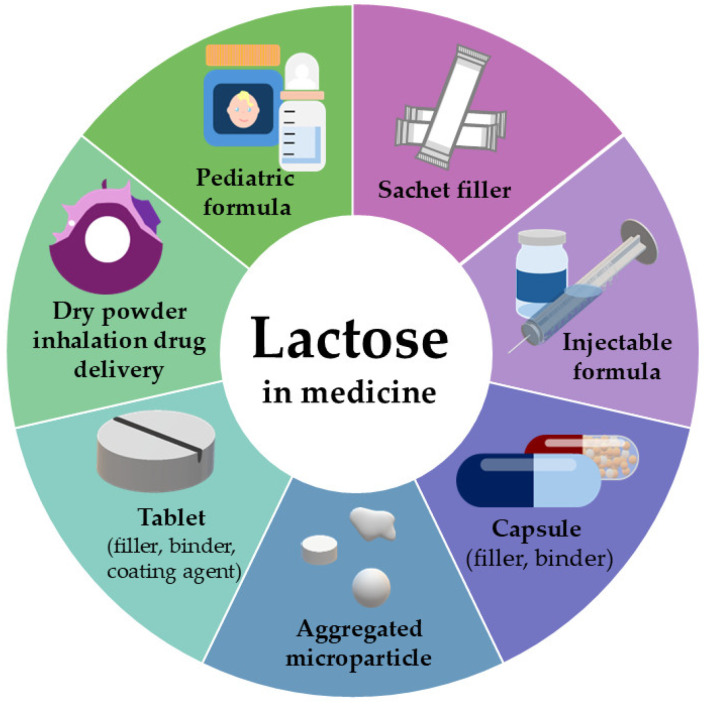
Lactose-containing formulas.

The substrate of the enzyme lactase, lactose, may enter the human body not only through dietary intake but also, albeit in relatively small quantities, via various medicines. Lactose is present as an excipient in approximately 60–70% of approved pharmaceutical products across different drug delivery systems [34]. As illustrated in Figure 3, formulators and manufacturers employ lactose in a wide range of dosage forms [35,36]. The reason is its favorable physical and chemical properties, including chemical inertia, stability, and non-toxicity, as well as its moderate cost [37]. Given its organoleptic characteristics, being white, odorless, and sweet-tasting, lactose is widely accepted in pharmaceutical formulations (Figure 3).

There are numerous ways to use lactose as an excipient, and it is present in various pharmaceutical forms. In the preparation of tablets and granules, it is frequently utilized as a filler. Lactose is also utilized by excipient manufacturers in the production of co-processed excipients, and spray-dried solely lactose particles are commercially available. The majority of lactose-based co-processed excipients, as well as the spray-dried (SD) forms, are amenable to direct compression, permitting tablet production without the necessity for preliminary operations (e.g., granulation), thereby streamlining the manufacturing process [38,39]. Additionally, these types of excipients can be utilized in the formulation of orally dispersible tablets, further expanding their applicability in modern pharmaceutical technology development [40,41].

Moreover, lactose functions as a cryoprotective excipient during the freeze-drying process due to its adsorption properties, protecting drugs against moisture [42]. It is also used as a cross-linker in gelatin hydrogel, forming a rigid layer that provides mechanical strength and protection to the dressing [42,43]. In Dry Powder Inhaler (DPI) formulations, lactose is often used as a carrier. However, lactose carrier particles are too large to penetrate deep into the respiratory system, resulting in most lactose depositing in the oropharynx. If swallowed, lactose can reach the gastrointestinal tract, causing intolerance effects in individuals with lactose intolerance [44].

Although the amount of lactose in a single dose of medication is well below the tolerable maximum, there can be significant individual differences [45]. However, predicting changes in lactose intake based on the digestive process is challenging due to varying rates of gastric emptying, pH levels, and intestinal motility [46].

Lactose, as a pharmaceutical excipient, is generally regarded as non-toxic and safe. However, adverse effects may occur in patients or users with pre-existing conditions such as malabsorption disorders, galactosemia, diabetes mellitus, or hypersensitivity to lactose. In accordance with all of the above and the EMA guidelines, users of medicinal products should be specifically warned if the medicinal product administered orally, parenterally or by inhalation contains lactose [34,47].

### 1.4. Characterization of Lactase Enzyme (Physical–Chemical Properties) and Industrial Applications

#### 1.4.1. β-Galactosidases

**Figure 4:**
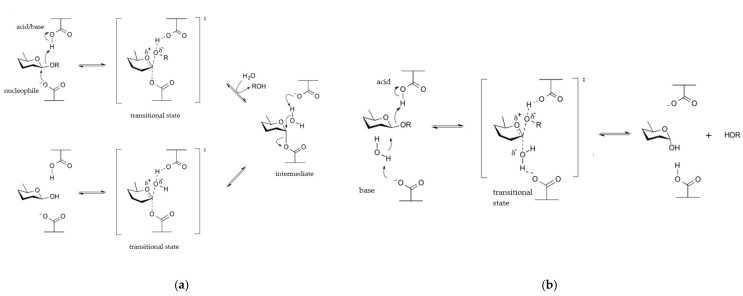
Reactions of retaining (**a**) and inverting (**b**) types of glycoside hydrolases (including the [transitional states]).

β-Galactosidases belong to the glycoside hydrolase enzyme family. These enzymes predominantly break down the disaccharide lactose (Figure 4) but can also catalyze the formation of various galacto-oligosaccharides. Additionally, β-galactosidases hydrolyze various aglycons, such as oNPG, commonly used to test enzyme activity [48,49]. Both prokaryotic microorganisms and eukaryotic organisms produce different β-galactosidases.

β-Galactosidase is crucial in many physiological processes, such as releasing stored substances. In mammals and bacteria, it provides energy for rapid growth through lactose hydrolysis, with free galactose playing a significant role in the metabolic recycling of galactolipids and glycoproteins [50]. In medicine and the food industry, the most important and commonly used enzymes are isolated primarily from yeasts like *K. lactis*, *K. fragilis*, *K. marxianus*, and *C. kefyr* and fungi such as *A. niger* or *A. oryzae*, but they can also be obtained from bacteria (*E. coli*) [51,52]. Enzymes with β-galactosidase activity belong to the GH 1, 2, 35, and 42 families [53]. Most β-galactosidases are part of the GH2 family, characterized by retaining β-galactosidases with narrow substrate specificity, reacting only with lactose and β-1,3 and β-1,6 galactosides [54].

#### 1.4.2. Lactase Enzyme

**Figure 5:**
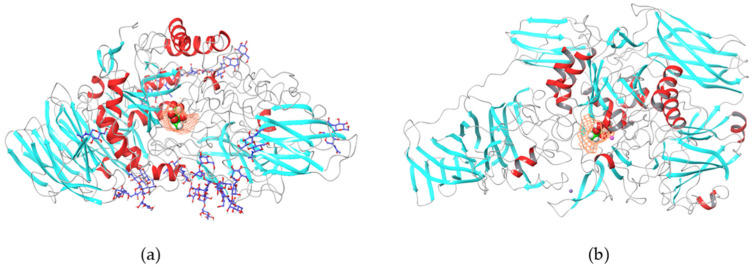
Three-dimensional model of the β-galactosidase enzyme from *A. oryzae*, 123 kDa (**a**), and *K. lactis*, 135 kDa (**b**), during substrate binding. Created after calculations by Schrödinger software ver. 11.15 (release 2019-2) in Maestro (version 12.0.012) based on the amino acid sequence of the enzyme.

Enzyme molecules such as lactase have many different structural variants depending on their source, with distinct properties and resistance to external effects (pH, temperature, etc.) [22]. Biochemically, β-galactosidase follows Michaelis–Menten kinetics, with substrate affinity (K_m_) for lactose typically 1–5 mM in fungal sources like *A. oryzae* (K_m_ = 1.899 mM and V_max_ = 1.044 µmol/min), indicating efficient binding in high-substrate industrial environments, compared to higher K_m_ (~10 mM) in yeast like *K. lactis*, which may require more enzymes for saturation. Stability is pH-dependent: *A. oryzae* variants are stable at pH 3–6 and temperatures up to 55 °C, minimizing denaturation during pasteurization or gastric transit, while *K. lactis* is optimal at pH 6–7 and <45 °C, limiting industrial versatility. These properties enhance process efficiency, reducing costs in lactose hydrolysis for dairy (e.g., 90% hydrolysis in 24 h at 37 °C) [55,56].The term lactase enzyme is a collective name covering a subclass of β-galactosidases. Generally, lactase enzyme refers to β-d-galactosidase-galactohydrolase, which belongs to the EC 3.2.1.23 subgroup [57]. In mammals, β-lactase is produced in the mammary glands, pancreas, and jejunal epithelial cells. Its primary function in the human body is to catalyze the hydrolysis of galactosyl from lactose. It also has known transgalactosylation activity, synthesizing galacto-oligosaccharides [58]. Lactases are produced in the intestines of mammals and more advanced microorganisms [59]. In medicinal products and food supplements, lactases are derived exclusively from the fungal species *A. oryzae* (~123 kDa) and *K. lactis* (~135 kDa) (Figure 5).

## 2. Materials and Methods

### 2.1. Systematic Search

This systematic review was conducted in accordance with the Preferred Reporting Items for Systematic Reviews and Meta-Analyses (PRISMA) guidelines [60]. The review protocol is registered in the Prospero database with ID CRD420251170582.

#### 2.1.1. Eligibility Criteria

The following criteria were established for articles to be eligible for inclusion in this systematic review: Only original research articles and reviews published in peer-reviewed journals were included, while editorials, conference papers, and commentaries were excluded. The search was confined to articles published in English between 1895 and 2025. Only β-galactosidase-containing oral dosage forms for enzyme replacement therapy were accepted.

#### 2.1.2. Search Strategy

A systematic search was conducted in PubMed, Scopus, ScienceDirect and Web of Science to identify relevant articles on formulations related to the β-galactosidase enzyme using search keywords and their equivalent synonyms. We developed our own search queries as follows: (((“beta-galactosidase”) OR (lactase)) OR (“β-galactosidase”)) AND (formulation). Database scanning was conducted on the 24 June 2025. The results were synthesized and tabulated accordingly.

#### 2.1.3. Data Collection and Extraction

We used the PRISMA 2020 flow diagram to extract the most relevant data essential for synthesizing the results. First of all, results obtained from all databases were exported to the EndNote reference manager, and duplicate studies were removed. The rest of the articles were screened successfully based on the title, and after that, the rest of the articles were excluded based on the abstract. Then, all eligible articles were reviewed and analyzed. The reviewers then cross-verified the relevant articles against the inclusion criteria. The relevant information was collected and tabulated into the following variables: Dosage Form, Purpose of innovation, Conclusion, and Reference.

## 3. Results

### 3.1. Case Study: Diversity of Lactase Products in Hungary

In Table 1, the different lactase-containing products available in Hungary are summarized. The data highlights the wide range of formulations on the market. The analysis was conducted using lactase-containing products obtained personally from various points of sale, mostly pharmacies and drug stores in Hungary.

**Figure 6:**
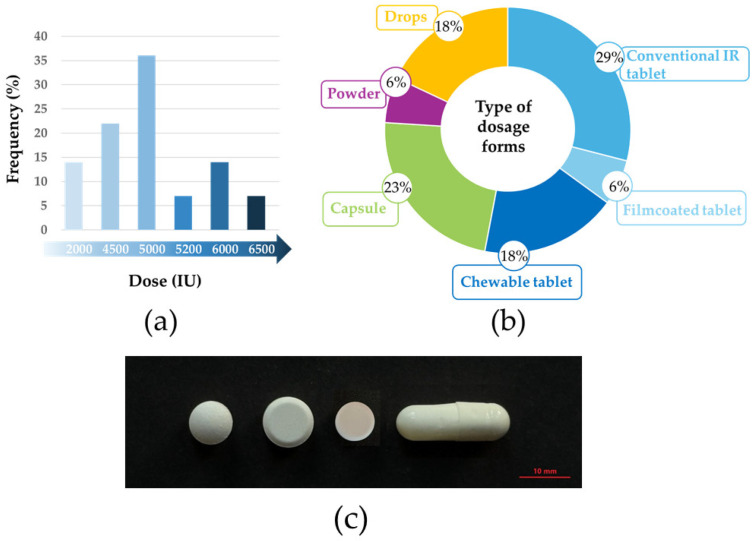
Distribution of different lactase enzyme doses (**a**), types of dosage forms (**b**) and examples of these dosage forms in physical form (**c**) marketed in Hungary.

The Hungarian market offers a wide range of lactase-containing products, including both medicinal preparations and dietary supplements in various forms such as chewable tablets, drops, powders, capsules, film-coated tablets, and conventional tablets (Figure 6). The only medicinal product identified is Lactase Strathmann, a chewable tablet containing the *A. oryzae* lactase enzyme. The majority of products are dietary supplements, with enzyme activities ranging from 2000 IU to 6500 IU per dose. Many formulations contain *A. oryzae* as the enzyme source, while a few also use *K. lactis*, and several do not disclose the source. These products are manufactured by both domestic companies such as Scitec Kft., BioTech USA Kft., and TEVA Gyógyszergyár Zrt. and international brands including PXG Pharma GmbH and Starlife s.r.o. The variety in dosage forms allows flexible use, from chewable options aimed at convenience to drops enabling adjustable dosing, reflecting the diverse needs of consumers with lactose intolerance.

Apparently most dietary supplements on the Hungarian market rely on increasing the amount of active ingredients, exceeding the recommended intake amount per dose. This approach offers a simple short-term solution, as higher enzyme doses aim to compensate for losses occurring in the stomach or during storage. However, its long-term effectiveness is uncertain, since quality control and clear source specification are often lacking, and overdosing does not ensure sustainable safety.

As shown in the first table and Figure 6, the vast majority of commercially available products are tablets, which are predominantly conventional (immediate release or IR) tablets, although film-coated tablets and chewable tablets are also available.

### 3.2. Database Search

**Figure 7:**
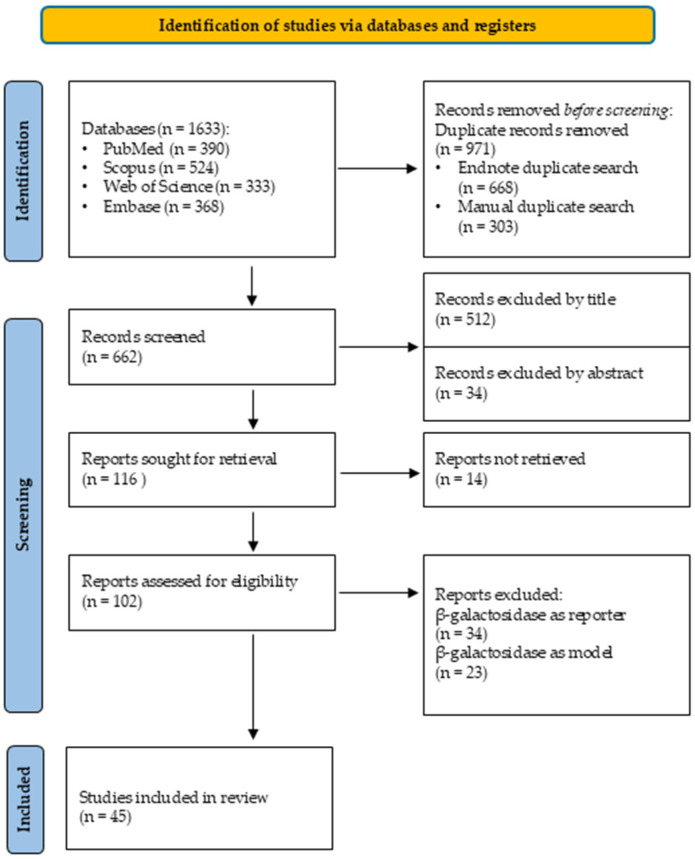
This PRISMA-2020 flow diagram shows the relevant articles included in this study.

For the systematic evaluation, a total of 1633 articles were retrieved through database searches. Among them, 390 were sourced from PubMed, 524 from Scopus, 333 from Web of Science and 368 from Embase. The identification and screening process is outlined in Figure 7.

During the search, we excluded all studies in which β-galactosidase was mentioned solely as a model enzyme [61], as a reporter protein in plasmid-based genetic modifications [62], as an enzyme involved in prodrug activation [63,64] or used in the dairy industry [65]. Consequently, 617 publications were excluded from the scope of our research after removing all the duplicates.

Table 2 summarizes recent advances in oral lactase enzyme delivery systems, highlighting innovations in dosage forms, materials, and release mechanisms. Technologies include pH-sensitive microparticles, nanogels, colloidal systems, double encapsulation, and targeted carriers such as halloysite nanotubes and wheat germ agglutinin-functionalized microspheres. Key strategies focus on enhancing enzyme stability in the gastrointestinal tract, controlling release profiles, and improving biological functions. Applications extend to oral protein therapeutics for lactose intolerance treatment and functional food development, demonstrating broad potential for improving enzyme-based interventions.

**Figure 8:**
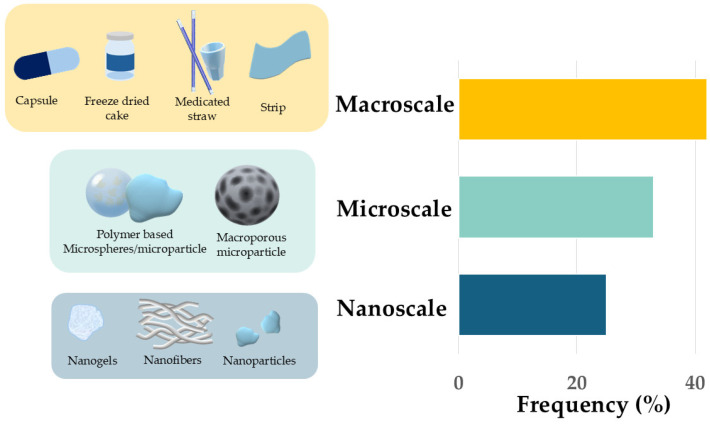
The size distribution of the different β-galactosidase-containing dosage forms found in the investigated articles.

Based on our selection criteria, Figure 8 illustrates different scales of β-galactosidase-containing oral drug delivery systems: macroscales formulation-like capsules, freeze-dried cakes, medicated straws, and strips), microscales (polymer-based microspheres and macroporous microparticles), and nanoscales (nanogels, nanofibers, and nanoparticles). In terms of frequency of occurrence, macroscale systems are the most dominant but the other two types are not far behind This indicates that although traditional macroscale formulations are currently prevalent, micro- and nanotechnologies hold significant potential for innovative, targeted, and more effective enzyme therapies.

## 4. Discussion

### 4.1. Applications of the β-Galactosidase Enzyme

The lactase enzyme is primarily used in the dairy industry to remove lactose from milk, producing lactose-free products for individuals with lactose intolerance. Additionally, its ability to catalyze transgalactosylation reactions is utilized to produce prebiotic galacto-oligosaccharides, which are used in probiotic foods. It is also added as an ingredient to prevent lactose crystallization during the production of sweeteners from whey syrup. Finally, lactase serves as an active ingredient in the treatment of lactose intolerance [52].

Commercially available lactases can come from various sources, each with different properties. The most common enzyme sources are filamentous fungi (*A. oryzae*), yeast (*K. lactis*), and bacteria (*B. circulans*, *E. coli*). Several factors can influence their activity. The enzyme derived from fungi shows higher activity in acidic environments and better heat stability than those from yeast. Enzymes from yeast and bacteria perform well in neutral pH and at lower temperatures. Therefore, in the dairy industry, due to the near-neutral pH of milk and chilled conditions, enzymes from yeast or bacteria are used. Some fungal lactases are most active in acidic conditions, making them unsuitable for milk’s pH. The pH optimum for *A. oryzae* lactase is 4.5, while other fungal lactases may have even lower pH optima [112]. Regarding temperature, different lactases exhibit pronounced variations in their optimal conditions for hydrolytic activity. Fungal lactases demonstrate elevated optimal temperatures for catalytic activity and structural stability, with maximal hydrolytic efficiency observed at 50–55 °C, but they also perform well at 37 °C. In contrast, yeast-derived neutral lactases undergo rapid denaturation above 40–45 °C; however, they retain catalytic activity at low temperatures, enabling effective application under refrigerated conditions [113]. Therefore, fungal-derived enzymes are used for lactose removal in acidic dairy products, such as sour cream, and their pH tolerance makes them suitable for pharmaceutical applications.

β-Galactosidase from *A. oryzae* continues to be the enzyme of choice for pharmaceutical and industrial applications compared to sources like *K. lactis*, due to its enhanced stability in acidic environments (optimal pH ~4.5), higher thermal tolerance (optimal 50–55 °C, maintaining activity at 37 °C), and generally recognized status as safe, making it ideal for oral formulations and acidic dairy processing. By contrast, the *K. lactis* enzyme performs best at neutral pH (~6.5–7) but rapidly loses activity above 40–45 °C and is more sensitive to gastric acids, limiting its use in therapeutic contexts. In addition, *A. oryzae* enables cost-effective large-scale fermentation with higher yields and lower immunogenicity concerns [114]. Sustainable enzyme production relies on renewable fungal or yeast strains like *A. oryzae*, optimizing fermentation to reduce water and energy usage by around 20%. Large-scale manufacturing also contributes to a circular economy by converting whey lactose into biofuels or sweeteners, thereby minimizing dairy waste [115].

### 4.2. Therapy for Lactose Intolerance

The direct therapy for lactose intolerance is the use of lactase-containing medications and dietary supplements, which usually contain one specific stream of lactase derived from *A. oryzae* (Yellow koji mold), also called Tilactase.

In addition to enzyme supplementation, clinical evidence supports adjunctive approaches aimed at enhancing lactose digestion and tolerance. Specific probiotic strains, such as *Lactobacillus* and *Bifidobacterium* species, as well as prebiotics like galacto-oligosaccharides (GOSs), have been shown to modulate the gut microbiome, improve lactose hydrolysis, and reduce gastrointestinal symptoms in lactose-intolerant individuals. Systematic reviews and randomized controlled trials indicate that probiotics can significantly improve symptom scores and breath hydrogen levels, although outcomes vary depending on strain, dose, and individual patient factors [116].

Taken together, these findings suggest that effective management of lactose intolerance may involve a combination of exogenous lactase supplementation and strategies that support endogenous lactase activity and gut microbiota modulation, tailored to the underlying etiology and individual patient response [117].

There may also be preparations containing or mixed with enzymes produced by *K. lactis*. The enzyme added to milk before consumption or taken with milk helps hydrolyze the lactose in the consumed food, significantly reducing the amount of hydrogen and other gases produced by the intestinal flora and thereby the different symptoms caused by them. Lactose hydrolyzing efficiency can vary depending on the source of the enzyme, even at the same dose. The extent of lactose hydrolysis is influenced not only by the administered enzyme dose but also by physiological factors such as gastric pH and bile acid concentration, which modulate the effectiveness of the exogenous enzyme. The recommended dosage provided on product packaging or in accompanying instructions is typically expressed in FCC units (Food Chemicals Codex Units), which denote the enzymatic activity per tablet. There are several lactase-containing products worldwide, not all of them well-characterized. The EFSA-recommended amount of lactose in a dose is 4500 FCCU [53]. Products typically contain 3000–6000 units per tablet, sufficient for consuming about 2–2.5 dl of milk or 10–12 g of lactose [118].

### 4.3. β-Galactosidase-Containing Pharmaceutical Formulations

Based on the formulations presented in Table 1 and Figure 6, we can conclude that fundamentally, to formulate the tablet form, manufacturers used conventional tableting excipients, namely various fillers, colloidal silicon dioxide as a glidant, and magnesium stearate most commonly used as a lubricant [119]. Among the fillers, it is important to note that lactose, a frequently used tableting excipient, cannot be used in these products. Therefore, manufacturers use water-insoluble but swelling material, such as microcrystalline cellulose (MCC), and also water-insoluble dicalcium phosphate (DCP), which provides excellent hardness to the tablets [119]. In the case of chewable tablets, a pleasant taste is also very important for their administration. For the chewable tablets listed in Table 1, in addition to flavoring agents, manufacturers use fillers that provide a sweet taste. Thus, in one formulation, the manufacturer uses xylitol as a filler, while in another (LactoMed), isomalt is used. The latter filler is often used in the confectionery industry to produce sugar-free products, but its use in the pharmaceutical industry is also growing [120].

In the case of powder dosage forms, compressibility is no longer a requirement—only the development of appropriate taste and flow properties necessary for packaging in sachets. Drops containing lactase are viscous liquids, where viscosity is increased by using glucose syrup or glycerin, for example. Increasing the viscosity of the liquid allows for accurate dosing, and glycerin is known to transform the native protein complex into a more compact state, preventing protein aggregation and thus increasing its stability [121]. All of the capsules in the table are hard capsules, and the capsule wall is made of gelatin.

Economic and regulatory factors substantially influence the accessibility and clinical adoption of lactase supplements, yet these aspects are often underrepresented in literature. The production of high-quality lactase, typically via microbial fermentation, requires considerable investment in strain development, downstream purification, and quality control. These costs are reflected in retail prices, which can limit affordability for certain patient populations, particularly in regions with limited healthcare coverage. Moreover, in many healthcare systems, lactase supplements are classified as dietary products rather than essential medicines and are not reimbursed, further restricting patient access.

Advanced delivery systems, such as enzyme-loaded nanoparticles, liposomes, or encapsulated microspheres, offer potential advantages including enhanced stability, controlled release, and improved bioavailability. However, these novel formulations are subject to rigorous regulatory investigation [122]. Authorities typically require the comprehensive characterization of physicochemical properties, enzyme activity under gastrointestinal conditions, and safety assessments including toxicity and immunogenicity. These regulatory challenges can delay or limit the translation of innovative lactase therapies into clinical practice.

Consequently, the combined impact of production costs, limited reimbursement, and regulatory complexity may constrain both patient access and the effective implementation of next-generation lactase delivery systems, despite their technological potential.

As a general rule, it is advisable to start treatment with a smaller dose and gradually increase the dosage. It is also recommended to take the dose just before or with the first bite of food and to repeat the dose if consuming dairy products again within 30–40 min. While significant issues are unlikely, the sugars produced during lactase breakdown can raise blood sugar levels, so caution is advised for diabetic patients [25].

Commercially available tablets or capsules can be effectively used as enzyme replacement therapy, and their efficacy has been confirmed by numerous studies. However, comparative studies [123] have shown that they can be more expensive than consuming pre-hydrolyzed lactose-free milk. In studies of preparations containing enzymes derived from *K. lactis*, non-toxicity was observed at an oral dose of 50 g/kg body weight [124]. Overall, the consumption of exogenous lactase with meals is effective, often practical, and safe [30].

### 4.4. Enzyme Delivery Systems

Examining Table 2 and Figure 8, it can be seen that different delivery systems for β-galactosidase have been explored to improve enzyme stability, activity, and applicability across food, pharmaceutical, and therapeutic fields.

Microsphere/macrosphere systems hydrogels and polymer beads like calcium-alginate, carrageenan, pectin and chitosan are widely used to encapsulate β-galactosidase to protect the enzyme from gastric acid and proteases, provide controlled release, and improve operational stability in food and oral-delivery contexts.

Nanocarrier-based systems, including organic (liposomes, polymeric nanoparticles, and micelles) and inorganic/hybrid nanocarriers (metal–organic frameworks and silica) have been explored to increase enzyme stability, offer enhanced protection from proteolysis, permit targeted delivery, reduce immunogenicity, and enable controlled release or tumor-targeted prodrug activation strategies.

Freeze-dried (lyophilized) powders are the standard to produce stable, solid enzyme formulations (oral powders, capsules, and ingredients for food processing). The proper selection of lyoprotectants (trehalose, sucrose, maltodextrin, etc.) and process parameters are critical to preserve enzyme structure and activity during drying and storage.

Each system differs in scalability, regulatory complexity, and mechanistic advantages, with microspheres and lyophilized powders being more commercially practical and nanocarriers offering the highest potential for specialized biomedical applications.

A comparison of lactase delivery systems demonstrates considerable variation in efficacy, stability, bioavailability, and commercial feasibility. Traditional formulations, such as polyacrylamide gels, freeze-dried cakes, and calcium-alginate pellets, provide moderate enzyme activity and protection against gastric conditions [76], while freeze-dried powders and polymer-based microparticles enhance storage stability. Microcapsules, casein matrix systems, and enteric-coated capsules improve targeted intestinal release and bioavailability. Advanced nanoscale and polymeric systems, including nanogels, alginate–chitosan microparticles, dextran nanoparticles, and polymeric nanocarriers, offer superior stability, controlled release, and high enzymatic activity, though manufacturing complexity and prices may limit commercial availability [125].

Innovative formats, such as orodispersible films, drinking straws [105] with enzyme pellets, and powders, combine enzyme protection with patient-friendly administration, broadening accessibility. Overall, while traditional systems are simpler and cost-effective, advanced delivery platforms provide enhanced performance and bioavailability, emphasizing the need to balance enzymatic efficacy, stability, targeted delivery, and suitability for practical clinical and commercial applications.

### 4.5. Future Directions

Forward-looking approaches include enzyme engineering via site-directed mutagenesis to enhance thermal stability (e.g., increasing half-life by 2× [126]), directed evolution for improved pH tolerance, and synthetic biology for recombinant production in engineered hosts like E. coli, yielding variants up to 30% higher activity [127]. They could optimize β-galactosidase for sustained-release formulations and broader therapeutics.

## 5. Conclusions

The development and production of biological medical products, or biologics, is becoming increasingly important in the pharmaceutical industry. Advancements in biotechnology over the past few years have allowed pharmaceutical companies to produce safer, cheaper enzymes with enhanced potency and specificity. Enzyme replacement therapy is a rapidly emerging branch of medicine with potential for treating a wide range of genetic disorders. In the field of enzyme replacement therapy, beta-galactosidase is a vital enzyme that plays a crucial role in the breakdown of complex sugars like lactose into simpler sugars. This enzyme deficiency is associated not just with lactose intolerance but some serious genetic disorders such as GM1 gangliosidosis and Morquio syndrome, highlighting its significance in certain metabolic pathways. The development of therapies involving β-galactosidase shows promise in addressing these disorders by supplementing the deficient enzyme in affected individuals.

In our study, we examined lactase-containing formulations currently available on the market. Our results show that traditional dosage forms (tablets, capsule, drops, and powder) are predominantly used, with more than half of the preparations being tablets (uncoated, filmcoated, and chewable). However, it is important to note that in a significant proportion of the publications we reviewed, the lactase carrier system is in the nano- or micro-size range, which may offer several advantages for patients.

In the development of lactase-containing preparations, two main approaches can be distinguished: the currently dominant market strategy of overdosing, which seeks to ensure efficacy by increasing the amount of active ingredients, and furthermore, the application of innovative pharmaceutical formulations that focus on enzyme stability, targeted release, and bioavailability. While the former provides a simple short-term solution, formulation innovation offers a more effective, safer, and sustainable therapeutic alternative, even at lower doses. In the future, the development of lactose intolerance therapies is expected to move toward intelligent, targeted release, and long-term stable formulations that achieve maximum efficacy with lower doses.

In the future, more research is expected to focus on developing more stable lactase-based formulas. One possible avenue is to identify and investigate enzyme variants with greater thermal, pH, or proteolytic stability that can be more effectively integrated into oral formulations in the long term. On the other hand, new excipients that are constantly appearing in the pharmaceutical and food industries offer further opportunities to improve lactase stability. The effect of these innovative carriers and stabilizers on lactase needs to be systematically evaluated in order to optimize the efficacy, durability, and patient-centered applicability of the formulations.

## Figures and Tables

**Table 1:** The different β-galactosidase-containing pharmaceutical products and dietary supplements found in the Hungarian market.

Brand Name	Manufacturer	Dosage Form	Dose (FCC)	Regulatory Classification	Source of Enzyme
Lactase	Strathmann (Hamburg, Germany)	chewable tablet	2900	medicine	*A. oryzae*
Coli comfort	Brand Up Pharma (Újlengyel, Hungary)	drops	143/drop (12 drops)	dietary supplement	*A. oryzae*, *K. lactis*
Lactase comfort	Brand Up Pharma (Újlengyel, Hungary)	drops	2500/drop (1 drop)	dietary supplement	no data
Co-lactase	Magnapharm Hungary (Budapest, Hungary)	drops	225/drop (2–4 drops)	dietary supplement	*A. oryzae*
Shake & Wait	Scitec Kft. (Budaörs, Hungary)	powder	2000	dietary supplement	*A. oryzae*
Antilact	BioTech USA Kft. (Budapest, Hungary)	capsule	4500	dietary supplement	no data
Laktáz Enzim	Scitec Kft. (Budaörs, Hungary)	capsule	5000	dietary supplement	*A. oryzae*
Laktáz enzim	Herbapharma Kft (Budapest, Hungary)	capsule	5000	dietary supplement	no data
Starlife Laktáz Enzim Star	Starlife s.r.o (Lidicka, Czechia)	capsule	5200	dietary supplement	*A. oryzae*
Mill & Joy	TEVA Zrt. (Debrecen, Hungary)	tablet	4500	dietary supplement	no data
Laktáz Enzim	Nutriversum Kft. (Budapest, Hungary)	tablet	5000	dietary supplement	no data
Biocom4You Laktáz Enzim	Ökonet Európa Kft. (Dabas, Hungary)	tablet	5000	dietary supplement	*A. oryzae*
LIVSANE Laktáz	PXG Pharma GmbH (Mannheim, Germany)	tablet	6000	dietary supplement	no data
JutaVit Laktáz	JuvaPharma Kft. (Felsőpakony, Hungary)	tablet	6500	dietary supplement	*A. oryzae*
Innolact	InnoPharm Kft. (Budapest, Hungary)	filmtablet	6000	dietary supplement	no data
Fermentált Laktáz	Natur Tanya Hungary Kft. (Budapest, Hungary)	chewable tablet	4500	dietary supplement	*A. oryzae*
LactaMed	Rubenza Kft. (Budapest, Hungary)	chewable tablet	5000	dietary supplement	no data

**Table 2:** Characteristics of different types of β-galactosidase-containing drug delivery systems.

Dosage Form	Purpose of Innovation	Conclusion	Reference
Polyacrylamide gel	Microcapsules with semipermeable enteric soluble materials	This complex microencapsulated lactase retained over 65% of its activity after 2 h of simulation in gastric juice.	Wang et al. (1993) [66]
Freeze-dried cake	Additive-based stabilization for increase enzyme stability during storage	Various excipients provide effective stabilization for β-galactosidase during storage.	Izutsu et al. (1994) [67]
Calcium-alginate based pellet	Immobilization with sodium alginate and calcium chloride in pellets to maintain protein stability and biological activity	The new pellet formulation effectively reduced symptoms and improved glucose absorption.	Xenos et al. (1998) [68]
Freeze-dried powder	Optimizing freeze-drying parameters without protectants to preserve protein activity after lyophilization	Protein activity preservation can be significantly improved by optimizing freezing and drying conditions, even without protectants.	Jiang & Nail (1998) [69]
Polymethylmethacrylate microparticles	Hydroxypropyl-β-cyclodextrin inhibits spray-drying-induced inactivation of β-galactosidase	Cyclodextrins can be useful for stabilizing excipients in the preparation of spray-dried protein pharmaceuticals.	Branchu et al. (1999) [70]
Thermo-sensitive xyloglucan-based gel	Thermally reversible gelation for sustained release	Xyloglucan-based gel may be suitable for sustained oral release.	Kawasaki et al. (1999) [71]
Gelatin–dextran-based hydrogel	Sol–gel transition, glycidyl methacrylate-dextran crosslinking via gamma irradiation for temperature-sensitive protein release	Sol–gel transition enables temperature-sensitive protein release; glycidyl methacrylate substitution and gelatin concentration influence the release profile.	Aso et al. (1999) [72]
Freeze-dried powder	Fast vs. slow cooling/heating process, sodium phosphate vs. potassium phosphate buffers to increase protein stability during freeze–thaw cycles	pH changes and cooling rate significantly affect protein activity recovery.	Pikal-Cleland et al. (2000) [73]
Microcapsules with different coatings	Microencapsulation for enzyme stability and controlled release	Propylene glycol monostearate and medium-chain triglyceride coatings effectively protect the enzyme and regulate release.	Kwak et al. (2001) [74]
Casein matrix microcapsules	Encapsulation with casein via emulsion polymerization, pH-sensitive release for targeted release	Microcapsules protected lactase from gastric acid and ensured controlled intestinal release.	Templeton et al. (2003) [75]
Lyophilized product	Polymers: polyvinyl alcohol, methylcellulose to increase stability, reducing aggregation	The Kohlrausch–Williams–Watts model describes aggregation/inactivation kinetics well; storage time can be extrapolated.	Yoshioka et al., (2003) [76]
Microencapsulated formulation	Microencapsulation with glycol monostearate and medium-chain triglyceride coating to enzyme release in small intestine	Microencapsulated β-galactosidase is effectively released in the gastro-intestinal tract.	Kim et al. (2006) [77]
Polysaccharide microparticles	Alginate core, poly(lactic-co-glycolic) acid shell (microencapsulation) for controlled release and enhanced protein stability	The double encapsulation system improved encapsulation efficiency and preserved activity.	Yuan et al. (2009) [78]
Poly(lactic-co-glycolic) acid microsphere	Reversible protein precipitation (with glycofurol and NaCl) for preserving protein stability during encapsulation	Reversible precipitation allowed enzyme preservation within poly(lactic-co-glycolic) acid microspheres without inactivation.	Giteau et al. (2008) [79]
Nano-coated lactose particle	Ultrasonically produced enzyme nanocoating for enzyme protection	Nanocoating remained stable for 1 month, with no loss of enzyme activity.	Genina et al. (2010) [80]
Two-phase capsule: gastric- and intestinal-active enzyme combination	Combination of enzymes from different sources and enteric coating for enhanced stability	The capsule hydrolyzed 3.5 times more lactose than the commercial product.	O’Connell & Walsh, (2010) [81]
Poly(lactic-co-glycolic) acid microparticles	Surface wheat germ agglutinin attachment, cross-linker: hexamethylene diamine and 1-ethyl-3(3-dimethylaminopropyl) carbodiimide	Targeted poly(lactic-co-glycolic) microparticles may be effective for long-term lactase supplementation.	Ratzinger et al. (2010) [82]
Poly(lactic-co-glycolic) acid microspheres	S/O/W emulsion, dextran-based core for controlled protein release, burst release reduction	The new microsphere production method reduces initial burst release and improves bioactivity.	Ren et al. (2011) [83]
Thermo-responsive nanogel (core cross-linked micelles)	Reversible Addition–Fragmentation chain Transfer (RAFT) polymerization, thermo-sensitive degradable core, poly(2-methacryloyloxyethyl phosphorylcholine) shell for protein encapsulation and controlled release	Nanogels prepared by RAFT are stable and biocompatible and effectively regulate protein release with temperature changes.	Bhuchar et al. (2012) [84]
Alginate–chitosan microparticles	Producing microparticles with chitosan, by a spray-drying process, for industrial applications	Microparticles effectively protect proteins from gastric acid and promote controlled intestinal release.	Estevinho et al. (2013) [85]
Dextran nanoparticles	Ionotropic gelation and drying for enzyme protection in the gastro-intestinal tract	Dextran-based formulation protected the enzyme in acidic environments and maintained its activity.	Wu et al., (2013) [86]
Gel discs (carrageenan + chitosan/polyethyleneimine)	Glutaraldehyde covalent immobilization for biotech and medical use	Different polyelectrolyte complex systems have varying effects on stability and kinetics.	Elnashar & Kahil (2014) [87]
Enteric-coated capsule	pH-sensitive polymer coating for enzyme protection from gastric acid	Enteric-coated capsules effectively protect the enzyme in the stomach and ensure its activity in the intestine.	He et al. (2014) [88]
Chitosan microcapsules	The influence of pH in the microencapsulation process, using a modified chitosan	The microencapsulated formulation was obtained at pH 6, being more than four times higher than the formulations produced with different pH levels.	Estevinho et al. (2015) [89]
Hydrogel (chitosan grafted)	Immobilization, controlled release for lactose-free food production	Chitosan-based grafted hydrogels are suitable for effective β-galactosidase immobilization and controlled release, reusable with stable activity.	Facin et al. (2015) [90]
Electrospun polymer fiber	Polyvinyl alcohol/polycaprolactone blend, addition of cryoprotectant (trehalose) for maintaining protein stability and biological activity	Electrospun fibers effectively preserve enzyme activity even when stored at room temperature.	Wagner et al. (2015) [91]
κ-carrageenan-based hydrogel beads	K^+^ ion stabilization, physical encapsulation for increased enzyme stability	Encapsulation increases activity and stability, but leakage may occur.	Zhang et al. (2016) [92]
pH-sensitive microparticles	Macroporous pH-sensitive Eudragit microparticle for targeted intestine release	The smart, pH-sensitive system provided enhanced protection and targeted drug release.	Kumar et al. (2017) [93]
Alginate–Ca(II) beads	Trehalose, arabic and guar gum additives for maintain protein stability and biological activity	Trehalose and guar gum improved stability; excipients influenced structure and heat stability.	Traffano-Schiffo et al. (2017) [94] Traffano-Schiffo et al. (2017) [95]
pH-sensitive, macroporous microparticle	Macropore formation, pH-sensitive response for small intestine targeting	The newly developed macroporous microparticles can be effectively used for directing proteins to the intestinal tract.	Homayun et al. (2018) [96]
Orodispersible films	Protein loaded orodispersible films (ODFs), based on blends of trehalose/pullulan by air- and freeze-drying.	The stability of β-galactosidase increased with increasing trehalose/pullulan ratios.	Tian et al. (2018) [97]
Powdered bulk granules	Spray-drying parameter optimization for more stable protein formulation	The precise optimization of drying conditions improves protein powder stability and solubility.	Lipiäinen et al. (2018) [98]
Lipid sponge	Formulation of a matrix for controlled delivery, to achieve a high protein load and to ensure high activity of the protein	The encapsulated β-galactosidase maintained its activity for a significantly longer time compared to the free solution at the same temperature.	Gilbert et al. (2019) [99]
Halloysite nanotube-embedded microparticle	Nanotube embedding, pH-sensitive for small intestine targeting	Halloysite nanotube systems provide significant stability benefits for oral protein administration.	Homayun et al. (2020) [100]
Colloidal systems (emulsions, liposomes, microgels)	Encapsulation, food-grade colloidal delivery systems for oral protein delivery and stabilization	Colloidal systems may be effective for the stable and targeted oral delivery of bioactive peptides and proteins.	Perry & McClements (2020) [101]
Nanocarrier (polymeric nanocarrier)	Polymer-based nanoparticle for improving enzyme stability	The formulation enhanced enzyme stability and bioavailability for oral administration.	Markwalter et al. (2020) [102]
Carboxymethylcellulose (CMC) microgel	Polymer matrix, pH-sensitive gelation for maintain enzyme activity and targeted release	CMC-based microgel improved enzyme stability and efficacy.	Silva et al. (2020) [103]
In situ gel-forming system (liquid dosage)	pH-sensitive gel-forming system for improving enzyme stability	The system provided excellent stability and biological activity for oral enzyme delivery.	Liu et al. (2022) [104]
Drinking straw filled with pellets	Novel oral administration method for children, elderly and those with dysphagia	This is an innovative child-friendly dosage form with powdered enzyme formulation in a straw.	Király et al. (2022) [105]
Nanocarriers	Nano-sized drug delivery systems provide protection, stability, and controlled release of proteins	Microfluidic mixing is an affordable and efficient platform for delivery of biological macromolecules.	Greco et al. (2023) [106]
Carboxymethyl chitosan– silica powder (biocatalyst)	One-pot silica gel route, maltose as lyoprotectant for enzyme stabilization	The biocatalyst showed high activity in the stomach (96%) and intestine (63%), is stable for 12 months and is non-cytotoxic.	Franco Tobón et al. (2023) [107] Franco Tobón et al. (2024) [108]
Lyophilized protein-liquid mixture with phytoglycogen	Addition of phyto glycogen dendrimers (PG1–PG16), focus on PG13 for protein stabilization during lyophilization	PG13 dendrimers are effective lyoprotectants and cake-forming agents for various proteins.	Park et al. (2024) [109]
Chitosan–alginate–pectin polymer gel	Polymer matrix formation and thermal stabilization for stabilization and bioavailability improvement	The combined gel system enhanced stability and enabled delivery in various environments.	Fraile-Gutiérrez et al. (2024) [110]
Polymer film	Mechanically triggered release for enzyme stabilization and controlled release locally	Mechanical activation enables homogeneous and controlled drug release, which is promising for local applications.	Bianco et al. (2024) [111]

## Data Availability

These data were derived from the following resources available in the public domain: [DrugBank and https://go.drugbank.com/; PubMed and https://pubmed.ncbi.nlm.nih.gov/; Scopus and https://www.scopus.com; Web of Science and http://www.webofscience.com; Science Direct and https://www.sciencedirect.com/].
